# The effectiveness of nonsteroidal anti-inflammatory agents in the treatment of pelvic inflammatory disease: a systematic review

**DOI:** 10.1186/2046-4053-3-79

**Published:** 2014-07-22

**Authors:** Divya Dhasmana, Emma Hathorn, Racheal McGrath, Anjum Tariq, Jonathan DC Ross

**Affiliations:** 1 Department of Genitourinary Medicine, Whittall Street Clinic, Whittall Street, Birmingham B4 6DH, UK; 2 Department of Genitourinary Medicine, The Fowler Clinic, New Cross Hospital, Wolverhampton WV10 0QP, UK

**Keywords:** nonsteroidal anti-inflammatory drugs, pelvic inflammatory disease, systematic review

## Abstract

**Background:**

Pelvic inflammatory disease (PID) is the result of infection ascending through the endocervix to the uterus and fallopian tubes. Inflammation driven by infected host cells appears to be central to the development of tissue damage and associated reproductive complications. Nonsteroidal anti-inflammatory agents (NSAIDs) therefore have the potential to reduce the sequelae associated with pelvic infection.

**Methods:**

A search of four electronic reference databases, an internet search for relevant grey literature and a review of the bibliographies of identified publications was used to identify studies evaluating NSAIDs in the management of PID. A predefined search strategy was used to identify studies that included women with PID aged over 16 and diagnosed after 1980. Randomized controlled trials, nonrandomized controlled trials, and cohort studies with comparison group data were included without language restriction. Two reviewers independently assessed the studies against agreed criteria and extracted relevant data using a standardized pro forma. A meta-analysis to calculate the relative risk associated with NSAID use was planned if appropriate.

**Results:**

Forty-three studies were identified. After reviewing abstracts or full texts, two randomized controlled trials were found to meet the selection criteria for inclusion. The use of NSAIDs was reported to improve tubal patency, reduce pelvic adhesions and reduce suprapubic pain but the studies were of poor quality with a high risk of bias. Meta-analysis of the data was not performed.

**Conclusions:**

Insufficient data is available to support or refute the efficacy of NSAIDs in the prevention of short or long-term complications of PID.

## Background

Pelvic inflammatory disease (PID) is an infectious and inflammatory disorder of the uterus, fallopian tubes and adjacent pelvic structures as a result of ascending infection from the endocervix. The highest prevalence is in 16- to 24-year-olds, reflecting the high rate of bacterial sexually transmitted infections found in this age group [1]. Complications include infertility, ectopic pregnancy and chronic pelvic pain, and can result in considerable physical and emotional morbidity, in addition to a significant financial burden on healthcare services.

*Chlamydia trachomatis* and *Neisseria gonorrhoeae* are the causative agents in approximately 40% of cases of PID [2]. *Mycoplasma genitalium*, *Trichomonas vaginalis*, *Herpes simplex* type 2, bacterial vaginosis-associated microorganisms and anaerobic organisms endogenous to the vaginal flora have also been isolated from the upper genital tract, although their role in the reproductive complications of pelvic infection remains unclear [3-8]. Inflammation driven by infected host cells appears central to the development of reproductive complications. Epithelial cells are the primary target of chlamydial infection, and are thought to initiate and sustain the host response through the secretion of chemokines, which recruit inflammatory leukocytes to the site of infection, and which also induce and regulate the inflammatory process. For example, Toll-like receptor-2 acts as a mediator of the innate immune response and has a role in both the production of inflammatory mediators and the development of upper genital tract pathology [9].

The adaptive immune response is also triggered by the release of inflammatory mediators. The CD4 Th-1 response is thought to be the main mechanism of infection resolution. Reinfection is common and it is proposed that an amplified Th-1 response to chronic or repeat infection might promote tissue damage and scarring. Chlamydia heat shock protein-60 (Chsp60) has been investigated as the possible cause of a delayed hypersensitivity reaction leading to a heightened inflammatory response. It has a highly conserved amino acid sequence, chlamydial and human sequences being 48% homologous. This similarity may result in an autoimmune response due to cross reactivity between self-hsp and Chsp-60. However, despite animal data supporting this hypothesis, the PID Evaluation and Clinical Health (PEACH) study did not demonstrate a correlation between increased antibody titres to Chsp60 and PID-related complications [10]. In contrast, antibodies to *Chlamydia trachomatis* elementary bodies, the extracellular form of *Chlamydia*, were independently associated with reduced rates of pregnancy and elevated rates of recurrent PID at 7 years of follow-up. It remains unclear whether chlamydial antibody titres simply reflect increased exposure, in terms of either chronicity of infection or reinfection, or if their presence represents a direct role in pathogenesis.

A number of host factors and cellular responses have been associated with susceptibility or protection from the development of reproductive complications. For example, the production of interferon-γ by peripheral blood mononuclear cells stimulated with Chsp60 has been strongly associated with protection against new *Chlamydia* infection [11]. Host factors that regulate the Th-1 response may also therefore be important in modifying the risk of long-term complications.

Current treatment guidelines focus on the eradication of infection using a regimen of broad-spectrum antibiotics [12,13]. The role of surgery is limited to the early division of adhesions and drainage of pelvic collections. No treatment is currently recommended to prevent or reduce the inflammatory process.

Nonsteroidal anti-inflammatory drugs (NSAIDs) are a group of analgesic drugs that act as nonselective inhibitors of the cyclooxygenase (COX) enzyme. The COX enzyme is required for the formation of prostaglandins that regulate the inflammatory response. There are two forms of this enzyme: a constitutive form, COX-1, that is present in the gastric mucosa, and an inducible form, COX-2; the latter form is preferentially expressed at sites of inflammation. Inhibition of the former leads to the well-described gastric side effects of NSAIDs, and inhibition of the latter to its therapeutic anti-inflammatory effects. Both COX-1 and COX-2 are expressed in the uterine epithelium at different times in early pregnancy [14], whilst COX enzymes are also known to be involved in endometrial pathology [15]. Animal studies have demonstrated that the immunoexpression of COX-2 was found to be strongest in endometrium with acute endometritis compared with normal endometrium or chronic endometritis [16]. In addition, COX-2 expression has been found to be an independent prognostic factor in uterine leiomyosarcomas [17].

The role of the COX enzyme in inflammation and adhesion formation in the murine model is well established and provides a mechanism for NSAIDs, through COX inhibition, to affect PID outcomes. Trauma to rat paws has been shown to induce expression of COX-2 and in turn increase the production of prostaglandin, resulting in oedema and a hyperalgesic response [18]. This pathway was blocked following the selective inhibition of COX-2 with indomethacin. Furthermore, following the induction of peritoneal fibrosis in rats, provision of NSAID was shown to reduce the extent and severity of adhesions in comparison with intraperitoneal saline or placebo [19].

Nonsteroidal anti-inflammatory drugs, commonly used as analgesics, may therefore provide a means of reducing the inflammation and subsequent fibrosis that leads to tubal damage associated with PID and its complications.

### Objectives

The objective of this review was to assess the clinical effectiveness of NSAIDs (in addition to antibiotic therapy) in the treatment of PID, including the prevention of long-term sequelae.

## Methods

### Search strategy

A search strategy was developed and used to identify relevant studies (Additional file 1). Databases were searched between March and May 2011 and again in June 2014 as follows: MEDLINE from PubMed 1980 to present, EMBASE 1980 to present, CINAHL 1981 to present and the Cochrane library. Searches were repeated in grey literature (Additional file 2) to identify any unpublished and ongoing research. No language restriction was applied.

Two reviewers independently searched each database, scanned titles and abstracts, and retrieved papers that fulfilled the predefined inclusion criteria, described next. In case of disagreement, a final decision on whether to include a study was made by consensus with a third reviewer.

### Types of study

Randomized controlled trials (RCTs), nonrandomized controlled trials and cohort studies reporting data from a comparison group were eligible for inclusion. Case series and case reports were excluded.

### Types of participant

Studies of females 16 years or over diagnosed clinically with PID as an inpatient or outpatient since 1980 were included.

### Types of intervention

Studies in which NSAIDs were given for the treatment of PID in combination with antibiotic therapy and where data were also reported from a comparison group were eligible for inclusion. The comparators included placebo or any intervention given in addition to antibiotic therapy for the treatment of PID, including paracetamol or opiates.

### Types of outcome

Short and long-term clinical markers of effectiveness were included.

Short term outcomes:

•Severity of pain.

•Time to resolution of pain.

•Length of inpatient stay.

Long-term outcomes:

•Chronic pelvic pain.

•Tubal patency.

•Tubal factor infertility.

•Ectopic pregnancy.

•Miscarriage.

### Data extraction

A standardized data extraction form was designed and, after internal review by the study team, utilized (Additional file 3). Two reviewers independently extracted data from the articles and disagreements were resolved following discussion. The authors of identified papers were contacted to request additional information where required. Study quality was assessed using the Cochrane Risk-of-Bias Tool [20].

### Data synthesis

Articles were stratified by type of NSAID and comparator. Key findings were reported.

## Results

Forty-three studies were identified by the search strategy. Thirty-six articles were excluded after title and abstract review, as they did not meet the predefined inclusion criteria. A further five articles were excluded after full text or abstract review of English translations (three trials did not include a comparator arm [21-23], one study compared the efficacy of two different NSAIDs [24] and one study did not give NSAIDs in addition to standard antibiotic therapy [25]). The remaining two studies were included for data extraction. References from these were reviewed for further relevant studies but none were identified.

### Study design

The two studies included for data extraction were RCTs. Bassil *et al.*[26] randomized 40 women with acute salpingitis or PID to receive piroxicam, a nonselective COX inhibitor, or no NSAID in addition to antibiotic therapy. Goffi *et al.*[27] randomized 42 women with mild acute PID to receive fentiazac, a new NSAID, or placebo in addition to antibiotic therapy. The duration of follow-up was short in both studies (9 to 11 weeks [26] and 10 days [27]).

### Quality

A risk-of-bias study is outlined in Additional file 4. The randomization process was not described clearly in either of the included studies. The nature of blinding of the investigator or subject to the intervention received was not clearly stated in either study. Outcome measures were not clearly defined and were often subjective. The basis of some statistical calculations in both trials was not reported, with only a *P* value provided. No additional information on study methodology, results or analysis was received following approaches to the study investigators.

### Outcomes

The chosen outcome measures were different in each study. Bassil *et al.*[26] performed a repeat laparoscopy at 9 to 11 weeks after the start of treatment and evaluated residual inflammation on inspection, peritoneal cytology, histological study of adhesions and tubal patency (categorized as mild, moderate or severe). These findings formed the basis of a subjective prognosis regarding fertility for each woman. In contrast, the primary outcome reported by Goffi *et al*. [27] was subjective resolution of adnexal pain on bimanual examination, or suprapubic and iliac fossa pain on palpation.

The key findings from the included studies are summarized in Table 1. A meta-analysis was not performed, owing to the heterogeneity and low methodological quality of the identified studies.

**Table 1:** Summary of included studies

	**Study design**	**Population**	**Size**	**Antibiotics**	**Control**	**Intervention**	**Analysis**	**Key findings**
[26]	RCT	Acute salpingitis or PID	40	Co-amoxiclav 1 g three times daily doxycycline 200 mg once daily intravenously for 5 days followed by co-amoxiclav 500 mg three times daily for 15 days and doxycycline 200 mg once daily for 21 days	No NSAID	Piroxicam 20 mg/day from day 3 post-operation for 25 days	Per protocol	Tubal patency: in severe PID, bilateral patency was seen in 1/7 (14.2%) of placebo group versus 7/9 (77.8%) of intervention group (*P* = 0.02, Fisher’s exact test)
		Confirmed by laparoscopy						Residual adhesions: in severe PID, more patients in intervention group had no residual adhesions, 6/9 (66.7%), versus control group, 1/7 (14.3%) (*P* = 0.06, Fisher’s exact test)
								No difference between arms in tubal patency or residual adhesions for mild or moderate PID
[27]	RCT	Mild acute PID	42	Tetracycline 500 mg four times daily for 10 days	Placebo	Fentiazac 200 mg twice daily for 7 days	Intention to treat	Suprapubic pain: resolution of pain occurred by day 7 in 9/21 (43%) of patients in the intervention group versus 5/21 (24%) in the control group (*P* = 0.2, *χ* square)
		Clinical diagnosis						
								Reduction in overall signs and symptoms: greater reduction in average score for severity of signs and symptoms in the intervention compared with the placebo group (figures providing the basis of the calculation not provided)
								Nausea reported in 4/21 patients receiving fentiazac (1 discontinuation) versus 2/21 in the control group (no discontinuations)

NSAID nonsteroidal anti-inflammatory drug; PID pelvic inflammatory disease; RCT randomized controlled trial.

## Discussion

### Effectiveness of NSAIDs

The studies included in this review provide insufficient data to support the use of NSAIDs in the prevention of long-term complications of PID. The use of NSAIDs was reported as reducing tubal obstruction, residual adhesions, pain and overall symptoms but the studies had limited power and were of low quality.

A role for NSAIDs in the reduction of inflammation and prevention of adhesions, through COX-2 inhibition, has been demonstrated in animal models but there is no comparable data for their effectiveness in altering the clinical course of inflammatory conditions in human subjects. For example, there is no evidence to suggest that NSAIDs modify the course of rheumatoid arthritis, where their effect is limited to symptomatic benefit [28]. Furthermore, the regular use of NSAIDs has to be balanced against their significant adverse effects including gastrointestinal haemorrhage, renal impairment and interactions with other commonly prescribed medication.

Inflammation appears to be central to the development of complications associated with PID and therefore other agents that modulate the immune system, including corticosteroids and biological immune modulators, might have a role in their prevention. Sanfilippo [29], in a case control study that involved incision and eversion of rat proximal uterine horns, treated the animals pre- and postoperatively with either corticosteroid or normal saline injections. Betamethasone phosphate produced a significant reduction in fibrosis when compared with all other corticosteroids or control solutions.

### Quality of included studies

A small number of individuals were included, with a total of only 82 patients identified from two studies. The marked differences between trials in antibiotic regimen used, treatment intervention and outcome measures meant that it was inappropriate to pool data for analysis. Both studies were described as RCTs but neither described their method of generating a random allocation sequence, their method of allocation concealment or who was blinded to the allocation schedule. A single operator was used to perform the second-look laparoscopies in the Bassil study [26] in an attempt to reduce inter-observer variability in reporting. It is not stated, however, whether the operator was blinded to the intervention received. In the Goffi study [27], there was no description of the placebo drug and how it compared in appearance and method of administration to fentiazac. The pain assessment was not validated and, in view of the subjective nature of pain and its reporting, inadequate blinding may have introduced bias.

The antibiotic therapy regimen given to women varied considerably between studies and was suboptimal when compared with current management guidelines. European and American guidelines recommend the use of broad-spectrum antibiotics to cover *Neisseria gonorrhoeae*, *Chlamydia trachomatis*, and aerobic and anaerobic bacteria commonly isolated from the upper genital tract. It is therefore unclear to what extent a suboptimal antibiotic regimen might have contributed to the reported clinical outcomes, including the persistence of pain and inflammation, although if randomization was robust then this should not have introduced systematic bias. The potential for bias is summarized in Additional file 4.

Patients included in the Bassil study [26] underwent an initial laparoscopy during which their presenting diagnosis was confirmed. This initial procedure enabled adhesiolysis, drainage of abscesses and peritoneal cavity lavage to be performed. Bessil *et al.*[26] concluded that a significantly greater number of those with severe PID treated with NSAIDs had fewer residual adhesions and a higher rate of tubal patency at second-look laparoscopy. Whilst the interventions performed during the initial laparoscopy were undertaken in both intervention and control groups, this would not be routinely performed in clinical practice and may limit the generalizability of the results.

Goffi *et al.*[27] reported a significant reduction in suprapubic pain in the group receiving a NSAIDs, compared with the control group. However, the basis of the statistical calculation was not clear from the data presented.

### Strengths and weaknesses

This is the first systematic review of the effectiveness of NSAIDs in the treatment of PID. A comprehensive literature review including grey literature and unrestricted by language was performed but it remains possible that relevant studies were missed. All relevant identified articles were obtained. The review complied with and is reported according to PRISMA guidelines (Additional file 5). English translations were obtained for foreign language papers, including both studies included in the analysis, and a number of studies were excluded based on translations of abstracts.

## Conclusions

There is insufficient data to support a recommendation for routine use of NSAIDs in the management of PID to reduce inflammatory complications. Further RCTs incorporating currently recommended antibiotic regimens with objective short and long-term outcome measures are needed to inform any change in clinical practice.

### Key findings

Inflammation is considered central to the development of acute and chronic reproductive complications of PID.

NSAIDs are used in the management of PID. Animal studies indicate that NSAIDs may reduce inflammation and fibrosis.

There is insufficient data to support or refute the efficacy of NSAIDs in preventing the complications of PID.

## Abbreviations

COX: cyclooxygenase; NSAID: nonsteroidal anti-inflammatory agent; PEACH: PID Evaluation and Clinical Health; PID: pelvic inflammatory disease; PRISMA: preferred reporting items for systematic reviews and meta-analyses; RCT: randomized controlled trial.

## Competing interests

The authors declare that they have no competing interests.

## Authors’ contributions

JDCR conceived the idea for study, analyzed the data and helped draft the manuscript. DD performed the literature search, analyzed the data and drafted the overall manuscript. EH performed the literature search, analyzed the data and helped draft the manuscript. RM translated foreign language articles and helped draft the manuscript. AT helped draft the manuscript. All authors read and approved the final manuscript.

## Supplementary Material

Additional file 1 Search strategy. Click here for file

Additional file 2 Grey literature search. Click here for file

Additional file 3 Data extraction form. Click here for file

Additional file 4 Risk-of-bias summary. Click here for file

Additional file 5 PRISMA checklist. Click here for file
