# Supplementary material for: The effectiveness of nonsteroidal anti-inflammatory agents in the treatment of pelvic inflammatory disease: a systematic review
Source: Syst Rev. 2014 Jul 22;3:79. doi: 10.1186/2046-4053-3-79 (PMC4125595; doi:10.1186/2046-4053-3-79)
Supplement: Additional file 1 — Search strategy. [file 2046-4053-3-79-S1.doc]

**Additional file 1: Search strategy**

1. exp PELVIC INFLAMMATORY DISEASE

2. exp ADNEXITIS

3. exp OVARY INFLAMMATION

4. Oophoritis.ti.ab

5. Parametritis.ti,ab

6. exp SALPINGITIS

7. exp ENDOMETRITIS

8. PID.ti.ab

9. exp ADNEXA DISEASE

10. exp NONSTEROIDAL ANTIINFLAMMATORY AGENT

11. NSAIDS$.ti,ab

12. 1 OR 2 OR 3 OR 4 OR 5 OR 6 OR 7 OR 8

13. 10 OR 11

14. 12 AND 13

15. 14 [Limit to: Publication Year 1980-Current and Human and Female and (Human Age Groups Adult 18 to 64 years or Aged 65+ years)]

16. 9 AND 13

17. 16 [Limit to: Publication Year 1980-Current and Human and Female and (Human Age Groups Adult 18 to 64 years or Aged 65+ years)]

18. 1 OR 2 OR 3 OR 4 OR 5 OR 6 OR 7

19. 13 AND 18

20. 19 [Limit to: Publication Year 1980-Current and Human and Female and (Human Age Groups Adult 18 to 64 years or Aged 65+ years)]
