# Supplementary material for: The effectiveness of nonsteroidal anti-inflammatory agents in the treatment of pelvic inflammatory disease: a systematic review
Source: Syst Rev. 2014 Jul 22;3:79. doi: 10.1186/2046-4053-3-79 (PMC4125595; doi:10.1186/2046-4053-3-79)
Supplement: Additional file 2 — Grey literature search. [file 2046-4053-3-79-S2.doc]

**Additional file 2: Grey Literature** **Search**

Aggressive Research Intelligence Facility (ARIF), Birmingham University

British Library

Clinical Trials.gov

Cochrane Central Register of Controlled Trials

Current Controlled Trials

Google Scholar

Open Grey

UK Clinical Research Network
