# Supplementary material for: The effectiveness of nonsteroidal anti-inflammatory agents in the treatment of pelvic inflammatory disease: a systematic review
Source: Syst Rev. 2014 Jul 22;3:79. doi: 10.1186/2046-4053-3-79 (PMC4125595; doi:10.1186/2046-4053-3-79)
Supplement: Additional file 3 — Data extraction form. [file 2046-4053-3-79-S3.doc]

**Additional file** 3: Data extraction form

| **General information** |  |
| --- | --- |
| Researcher |  |
| Date of extraction |  |
| Record number |  |
| Author |  |
| Article title |  |
| Country of origin |  |
| Source of funding |  |
|  |  |
| **Study characteristics** |  |
| Aim/Objective |  |
| Study design |  |
| Randomisation |  |
| Blinding |  |
| Inclusion criteria |  |
| Exclusion criteria |  |
| Number randomised |  |
| Number analysed |  |
| Duration of follow-up |  |
|  |  |
| **Participant characteristics** |  |
| Age |  |
|  |  |
| **Intervention** |  |
| Standard treatment (antibiotics) |  |
| Intervention |  |
| Control |  |
|  |  |
| **Outcome data/Results** |  |
| Primary outcome |  |
| Secondary outcome |  |
|  |  |
| **Notes** |  |
