# Supplementary material for: The effectiveness of nonsteroidal anti-inflammatory agents in the treatment of pelvic inflammatory disease: a systematic review
Source: Syst Rev. 2014 Jul 22;3:79. doi: 10.1186/2046-4053-3-79 (PMC4125595; doi:10.1186/2046-4053-3-79)
Supplement: Additional file 4 — Risk-of-bias summary. [file 2046-4053-3-79-S4.doc]

**Additional file4: Risk of bias summary**

Blinding of participants and personell

Blinding of outcome data

Incomplete outcome data addressed addressed

Random sequence generation

Allocation concealment

Free of selective reporting

| High | high | - | + | ? | ? |
| --- | --- | --- | --- | --- | --- |
| High | high | + | + | ? | ? |

Bassil 1991

Goffi 1989

Key:

+ Yes;

- No;

? Unclear – insufficient information given to judge

High High risk of bias

Low Low risk of bias
